# Effectiveness and safety of structured exercise vs. no exercise for asymptomatic aortic aneurysm: systematic review and meta-analysis

**DOI:** 10.1590/1677-5449.190086

**Published:** 2020-05-08

**Authors:** Ricardo de Ávila Oliveira, Eliza Nakajima, Vladimir Tonello de Vasconcelos, Rachel Riera, José Carlos Costa Baptista-Silva

**Affiliations:** 1 Universidade Federal de Uberlândia – UFU, Departamento de Cirurgia, Uberlândia, MG, Brasil.; 2 Universidade Federal de São Paulo – UNIFESP, Departamento de Medicina, São Paulo, SP, Brasil.

**Keywords:** aortic aneurysm, abdominal aortic aneurysm, exercise, postoperative complications, aneurismas de aorta, aneurismas de aorta abdominal, exercícios, complicações pós-operatórias

## Abstract

We conducted a systematic review to compare the effectiveness and safety of exercise versus no exercise for patients with asymptomatic aortic aneurysm. We followed the guidelines set out in the Cochrane systematic review handbook. We searched Medline, Embase, CENTRAL, LILACS, PeDRO, CINAHL, clinicaltrials.gov, ICTRP, and OpenGrey using the MeSH terms “aortic aneurysm” and “exercise”. 1189 references were identified. Five clinical trials were included. No exercise-related deaths or aortic ruptures occurred in these trials. Exercise did not reduce the aneurysm expansion rate at 12 weeks to 12 months (mean difference [MD], −0.05; 95% confidence interval [CI], −0.13 to 0.03). Six weeks of preoperative exercise reduced severe renal and cardiac complications (risk ratio, 0.54; 95% CI, 0.31–0.93) and the length of intensive care unit stay (MD, −1.00; 95% CI, −1.26 to −0.74). Preoperative and postoperative forward walking reduced the length of hospital stay (MD, −0.69; 95% CI, −1.24 to −0.14). The evidence was graded as ‘very low’ level.

## Resumo

Foi realizada revisão sistemática para comparar a efetividade e a segurança de exercícios *versus* não exercícios em pacientes assintomáticos com aneurisma de aorta. Usamos os termos MeSH *aortic aneurysm* e *exercise* para as bases MEDLINE, Embase, CENTRAL, LILACS, PeDRO, CINAHL, clinicaltrials.gov, *International Clinical Trials Registry Platform* (ICTRP) e OpenGrey. Foram obtidas 1.189 referências. Cinco ensaios clínicos foram incluídos. Não houve morte ou rotura associada ao exercício. Além disso, este não reduziu a velocidade de crescimento do aneurisma em 12 semanas a 12 meses [diferença de médias (DM) −0,05; intervalo de confiança de 95% (IC95%) −0,13 a 0,03]. Seis semanas de exercícios pré-operatórios reduziram complicações clínicas renais e cardíacas (razão de risco 0,54; IC95% 0,31–0,93) e a permanência em unidade de terapia intensiva (DM −1,00; IC95% −1,26 a −0,74). Caminhadas nos períodos pré e pós-operatório reduziram a permanência hospitalar. A evidência foi classificada como de muito baixa qualidade.

## INTRODUCTION

An aortic aneurysm is a permanent localized aortic dilatation that is at least 50% larger than the normal diameter.^1^^,^^2^ The estimated prevalence is 4.0% to 8.9% in men and 1.3% to 2.2% in women aged ≥55 years.^3^^,^^4^ Approximately 80% of aortic aneurysms are located in the abdominal aorta.^5^ They usually have an asymptomatic natural history and so diagnosis is made after thorough investigation.^6^ The most feared complication of aortic aneurysms is rupture, which leads to death in up to 90% of patients.^7^ The risk of rupture increases as the aneurysm diameter increases.^2^^,^^8^^,^^9^ Surgery is recommended when an aneurysm reaches 50 mm in women or 55 mm in men,^10^ because at this point the risks of surveillance outweigh the risks of surgery. No clinical interventions have been found to be effective for reducing the growth rate or risk of rupture before an aneurysm reaches these diameters.^10^^,^^11^

In one study, 87.7% of the patients diagnosed had aneurysms with diameters of <3.5 cm.^12^ Therefore, despite the tremendous effort that has been expended on surgical research, there is not enough information to recommend non-pharmacologic clinical treatment for most patients, other than smoking cessation and controlling blood pressure. Even among patients with small aneurysms, the most frequent cause of death is myocardial infarction and stroke, not aneurysm rupture.^10^^,^^12^ Moreover, when patients do undergo surgery, 41% of deaths are also related to cardiovascular events.^13^ Therefore, an aortic aneurysm is a risk factor for death and has a risk of mortality 50% higher than that in persons with no aortic pathology.^12^

Exercise and smoking cessation help to reduce mortality and improve quality of life.^14^^,^^15^ Exercise is a subgroup of physical activity defined as planned, structured, and repetitive activities performed with the objective of improving or maintaining physical fitness.^16^ Despite the importance of exercise, there is no consensus regarding exercise recommendations for patients with aortic aneurysms,^17^^,^^18^ because of the fear of aneurysm rupture and doubts about the effectiveness of exercise. Hence, a systematic review of the literature is crucial to describe the risks and benefits of exercise for patients with aortic aneurysms.

The study was performed to assess the effectiveness and safety of exercise for asymptomatic patients with an aortic aneurysm.

## MATERIALS AND METHODS

This review was conducted in the Post-graduate Program in Evidence-based Healthcare at the Universidade Federal de São Paulo (UNIFESP), São Paulo, SP, Brazil. It followed the recommendations contained in the Cochrane Handbook for Systematic Reviews of Interventions,^19^ and reporting of the results complies with the PRISMA Statement for quality in publication.^20^ The review protocol was registered on the PROSPERO database.^21^ The review was also approved by the institutional ethics committee (CAAE number: 57716016.0.0000.5505).

Randomized and quasi-randomized clinical trials were considered for inclusion. Due to the nature of the intervention, crossover studies were not considered for this review.

The inclusion criteria were sedentary patients (those performing only daily activities during the last year), adults (≥18 years of age), and the presence of an aortic aneurysm confirmed by a diagnostic imaging examination.

The exclusion criteria were rapid growth of aneurysms (0.5 cm within 6 months or 1.0 cm within 1 year), saccular aneurysms, complicated aneurysms (such as symptomatic, completely thrombosed, or ruptured aneurysms), and inflammatory and infectious aneurysms. High intensity interval training exercises were excluded.

Any exercise was considered (individual or in groups, assisted or self-managed, aerobic, stretching or strengthening; any intensity, frequency, and duration; and alone or combined with any other intervention), as long as the same co-intervention was also performed in the comparison group. This group of participants was designated the exercise group. For the purposes of the present study, exercise was defined as a subgroup of physical activity that is planned, structured, and repetitive and aims to improve or maintain one or more components of physical fitness.^16^ The comparators considered in this review were patients receiving no intervention and patients on a waiting list. If a study compared different types of exercises (e.g., strength exercises versus resistance exercises), we considered performing a comparison of exercises versus advice for exercising or a different type of exercise used. These patients were designated the no exercise group.

### Primary outcomes

- All-cause mortality in the short-term (up to 30 days after beginning exercise) and long-term (from 30 days to ≥1 year after starting exercise);
- Number of participants presenting with aneurysmal rupture;
- Aneurysm growth rate (change, in millimeters (mm), in the aneurysm diameter from baseline to the end of the study).

### Secondary outcomes

- Quality of life, measured by any validated tool;
- Number of participants referred for aneurysm surgery;
- Number of participants presenting with at least one severe short-term (up to 24 hours after surgery), intermediate-term (from 24 hours to 30 days after surgery), or long-term (>30 days after surgery) complication. A severe complication was defined as myocardial infarction, prolonged inotropic support, new-onset arrhythmia, unstable angina, postoperative pneumonia, unexplained re-intubation, or renal insufficiency (requirement for dialysis or a >20% reduction in creatinine clearance);
- Hospital stay related to aneurysm surgery (in days);
- Intensive care unit stay after aneurysm surgery (in days);
- Forced expiratory volume in 1 second as measured with a spirometer.

Any outcome not mentioned in the protocol was described as a non-proposed outcome in the results.

The following electronic databases were searched and updated: Literatura Latino Americana em Ciências da Saúde e do Caribe (LILACS) (via the Biblioteca Virtual em Saúde [BVS], from 1966 to 13 December 2018), Medline (via PubMed, from inception to 13 December 2018), Cochrane Central Register of Controlled Trials (CENTRAL) (via Wiley Cochrane Library, December 2018 Edition), Embase (via Elsevier, from 1974 to 13 December 2018), PEDro (via BVS, from inception to November 2018), and Cumulative Index to Nursing and Allied Health Literature (CINAHL) (EBSCO, from inception to 7 November 2018). Additional searches were conducted on the trial registry databases ClinicalTrials.gov, the World Health Organization International Clinical Trials Registry Platform (ICTRP) search portal, and the gray literature (http://www.opengrey.eu/) (from inception to 13 December 2018). A manual search was also performed of the reference lists of all studies included and relevant systematic reviews.

There were no search limits for data, status, or language of publication. The search strategy for Medline is shown in Table 1.

**Table 1:** MEDLINE search strategy.

**MEDLINE via PubMed search strategy**	(“Aortic Aneurysm”[Mesh] OR (Aortic Aneurysm) OR (Aneurysms, Aortic) OR (Aortic Aneurysms) OR (Aneurysm, Aortic)) AND ((“Exercise”[Mesh]) OR (Exercise) OR (Exercises) OR (Exercise, Physical) OR (Exercises, Physical) OR (Physical Exercise) OR (Physical Exercises) OR (Exercise, Isometric) OR (Exercises, Isometric) OR (Isometric Exercises) OR (Isometric Exercise) OR (Exercise, Aerobic) OR (Aerobic Exercises) OR (Exercises, Aerobic) OR (Aerobic Exercise) OR “Physical Fitness”[Mesh] OR (Fitness, Physical) OR (Physical Fitness) OR “Exercise Therapy”[Mesh] OR (Therapy, Exercise) OR (Exercise Therapies) OR (Therapies, Exercise) OR “Physical Exertion”[Mesh] OR (Exertion, Physical) OR (Exertions, Physical) OR (Physical Exertions) OR (Physical Effort) OR (Effort, Physical) OR (Efforts, Physical) OR (Physical Efforts) OR “Sports”[Mesh] OR (Sport) OR (Athletics) OR (Athletic) OR “Exercise Movement Techniques”[Mesh] OR (Movement Techniques, Exercise) OR (Exercise Movement Technics) OR (Pilates-Based Exercises) OR (Exercises, Pilates-Based) OR (Pilates Based Exercises) OR (Pilates Training) OR (Training, Pilates) OR “Physical Endurance”[Mesh] OR (Endurance, Physical) OR (Endurances, Physical) OR (Physical Endurances))

Two reviewers independently screened the titles and abstracts for selection and inclusion using Rayyan software.^22^ They also extracted data and assessed the methodological quality of the studies included as described in the PROSPERO registry database.^21^ A third reviewer resolved any disagreements at each stage.

The strategies for data synthesis, meta-analysis, effect size, subgroup, and sensitivity analysis are also described in the PROSPERO database.^21^ RevMan 5.3 software^23^ was used to measure the effect size and perform a meta-analysis when possible. A funnel plot was also planned as part of the protocol.

The GRADE approach was used to evaluate the quality of the body of evidence.^24^ Each decision to downgrade the quality of studies was justified (Tables 2, 3, 4, 5, and 6). A summary-of-findings table was created using GRADEpro GDT considering the primary outcomes and the main comparisons (exercise vs. no exercise at 7- to 12-week surveillance and at 3 years; exercise vs. no exercise before surgery; exercise vs. no exercise after surgery; and exercise vs. no exercise before and after surgery).^24^ The outcomes were death, aortic rupture, aneurysm growth rate, number of patients with at least one cardiovascular complication, and number of patients referred for surgery.

**Table 2:** GRADEpro-GDT judgment of the quality of evidence: GRADE question: Should exercise be indicated for patients with aortic aneurysms at surveillance?

**Summary of findings:**					
**Exercise compared to no exercise for aortic aneurysm patients at surveillance in intermediate term (7-12 w)**					
**Patient or population: aortic aneurysm patients at surveillance in intermediate term (7-12 w)**					
**Setting:**					
**Intervention: exercise**					
**Comparison: no exercise**					
**Outcomes**	**Anticipated absolute effects** ^*^ **(95% CI)**		**Relative effect (95% CI)**	**Nº of participants (studies)**	**Certainty of the evidence (GRADE)**
	**Risk with no exercise**	**Risk with exercise**			
Mortality follow up: range 7 weeks to 12 weeks	0 per 100	0 per 100 (0 to 0)	not estimable	263 (4 RCTs)	⨁◯◯◯ VERY LOW ^a,b,c,d^
Aortic rupture follow up: range 7 weeks to 12 weeks	0 per 100	0 per 100 (0 to 0)	not estimable	263 (4 RCTs)	⨁◯◯◯ VERY LOW ^a,b,c,d^
Aneurysm growth rate follow up: range 7 weeks to 12 weeks	The mean aneurysm growth rate was 0	The mean aneurysm growth rate in the exercise group was 0.06 lower (0.23 lower to 0.11 higher)	-	(2 RCTs)	⨁◯◯◯ VERY LOW ^b,c,d,e^
Number of patients with at least one cardiovascular complication follow up: range 7 weeks to 12 weeks	0 per 100	0 per 100 (0 to 0)	RR 100.00 (0.07 to 35.46)	263 (4 RCTs)	⨁◯◯◯ VERY LOW ^a,b,c,d^
Number of patients who reached threshold for surgery follow up: range 7 weeks to 12 weeks	0 per 100	0 per 100 (0 to 0)	not estimable	263 (4 RCTs)	⨁◯◯◯ VERY LOW ^a,b,c,d^

* The risk in the exercise group (and its 95% confidence interval) is based on the assumed risk in the comparison group and the relative effect of the intervention (and its 95% CI); CI: Confidence interval; MD: Mean difference; RR: Risk ratio; GRADE Working Group grades of evidence. High certainty: We are very confident that the true effect lies close to that of the estimate of the effect. Moderate certainty: We are moderately confident in the effect estimate: The true effect is likely to be close to the estimate of the effect, but there is a possibility that it is substantially different. Low certainty: Our confidence in the effect estimate is limited: The true effect may be substantially different from the estimate of the effect. Very low certainty: We have very little confidence in the effect estimate: The true effect is likely to be substantially different from the estimate of effect. Explanations:

a. Half of the studies did not have blinded outcome assessment or did not have allocation concealment or the randomization method was unclear;

b. The time point of measurement was not long enough to support any conclusions;

c. Low number of events;

d. Small sample size;

e. The study had unblinded outcome assessment and did not have allocation concealment. The randomization method was unclear and there were incomplete outcome data.

**Table 3:** GRADEpro-GDT judgment of the quality of the evidence: GRADE question: Should exercise be indicated for patients with aortic aneurysms at surveillance?

Summary of findings:					
Exercises compared to no exercise for aortic aneurysm patients at surveillance in long term (at 3 y)					
Patient or population: aortic aneurysm patients at surveillance in long term (at 3 y)					
Setting:					
Intervention: exercise					
Comparison: no exercise					
**Outcomes**	**Anticipated absolute effects** ^*^ **(95% CI)**		**Relative effect**	**Nº of participants**	**Certainty of the evidence (GRADE)**
	**Risk with no exercise**	**Risk with exercise**	**(95% CI)**	**(studies)**	
Mortality follow up: mean 3 years	0 per 100	0 per 100 (0 to 0)	not estimable	140 (1 RCT)	⨁◯◯◯ VERY LOW a,b,c,d,e
Aortic rupture follow up: mean 3 years	0 per 100	0 per 100	not estimable	45	⨁◯◯◯
		(0 to 0)		(1 RCT)	VERY LOW ^a,b,d,^e,f
Aneurysm growth rate	The mean aneurysm growth rate was 0.54	The mean aneurysm growth rate in the exercise group was 0.06 lower (0.23 lower to 0.11 higher)	-	45	⨁◯◯◯
follow up: mean 3 years				(1 RCT)	VERY LOW ^a,b,c,d,e^
Number of patients with at least one cardiovascular complication	0 per 100	0 per 100	not estimable	45	⨁◯◯◯
follow up: mean 3 years		(0 to 0)		(1 RCT)	VERY LOW ^a,b,c,d,e^
Number of patients who reached threshold for surgery	13 per 100	4 per 100	RR 0.31	140	⨁◯◯◯
follow up: mean 3 years		(1 to 15)	(0.09 to 1.11)	(1 RCT)	VERY LOW ^a,b,c,d,e^

* The risk in the exercise group (and its 95% confidence interval) is based on the assumed risk in the comparison group and the relative effect of the intervention (and its 95% CI). CI: Confidence interval; MD: Mean difference; RR: Risk ratio. GRADE Working Group grades of evidence. High certainty: We are very confident that the true effect lies close to that of the estimate of the effect. Moderate certainty: We are moderately confident in the effect estimate: The true effect is likely to be close to the estimate of the effect, but there is a possibility that it is substantially different. Low certainty: Our confidence in the effect estimate is limited: The true effect may be substantially different from the estimate of the effect. Very low certainty: We have very little confidence in the effect estimate: The true effect is likely to be substantially different from the estimate of effect. Explanations:

a. The randomization method, allocation concealment, blinding of outcome assessment, and other sources of bias are unclear. There is a high risk of incomplete outcome data;

b. The intervention was performed in a very controlled setting, not applied to the usual patient;

c. There was only one study;

d. The sample size is too small to make a judgment;

e. The number of events were small;

f. Single study. Large number of patients lost to follow-up.

**Table 4:** GRADEpro-GDT judgment of the quality of the evidence: GRADE question: Should exercise be indicated for patients with aortic aneurysms before surgery?

**Summary of findings:**						
**Exercises compared to no exercise for aortic aneurysm patients before surgery**						
**Patient or population: aortic aneurysm patients before surgery**						
**Setting:**						
**Intervention: exercise**						
**Comparator: no exercise**						
**Outcomes**	**Anticipated absolute effects** ^*^ **(95% CI)**		**Relative effect (95% CI)**	**Nº of participants (studies)**	**Certainty of the evidence (GRADE)**	**Comment**
	**Risk with no exercise**	**Risk with exercise**				
Mortality follow up: mean 30 days	3 per 100	3 per 100	RR 1.00	124	⨁◯◯◯	Mortality was related to surgery. There was no mortality related to exercise.
		(3 to 3)	(0.93 to 1.07)	(1 RCT)	VERY LOW ^a,b^	
Aortic rupture follow up: mean 30 days	0 per 100	0 per 100	not estimable	124	⨁◯◯◯	
		(0 to 0)		(1 RCT)	VERY LOW ^a,b^	
Aneurysm growth rate - not measured	-	-	-	-	-	
Number of patients with at least one cardiovascular complication follow up: mean 30 days	23 per 100	8 per 100	RR 0.36	124	⨁◯◯◯	
		(3 to 21)	(0.14 to 0.93)	(1 RCT)	VERY LOW ^a,b^	
Number of patients who reached threshold for surgery - not measured	-	-	-	-	-	

* The risk in the exercise group (and its 95% confidence interval) is based on the assumed risk in the comparison group and the relative effect of the intervention (and its 95% CI). CI: Confidence interval; RR: Risk ratio; GRADE Working Group grades of evidence. High certainty: We are very confident that the true effect lies close to that of the estimate of the effect. Moderate certainty: We are moderately confident in the effect estimate: The true effect is likely to be close to the estimate of the effect, but there is a possibility that it is substantially different. Low certainty: Our confidence in the effect estimate is limited: The true effect may be substantially different from the estimate of the effect. Very low certainty: We have very little confidence in the effect estimate: The true effect is likely to be substantially different from the estimate of effect. Explanations:

a. Due to nature of intervention, it was impossible to blind participants and personnel;

b. Single study, low number of events

**Table 5:** GRADEpro-GDT judgment of the quality of the evidence: GRADE question: Should exercise be indicated for patients with aortic aneurysms after surgery?

**Summary of findings:**					
**Exercise compared to no exercise for aortic aneurysm patients after surgery**					
**Patient or population: aortic aneurysm patients after surgery**					
**Setting:**					
**Intervention: exercise**					
**Comparison: no exercise**					
**Outcomes**	**Anticipated absolute effects** ^*^ **(95% CI)**		**Relative effect**	**Nº of participants**	**Certainty of the evidence**
	**Risk with no exercise**	**Risk with exercise**	**(95% CI)**	**(studies)**	**(GRADE)**
Mortality - not measured	-	-	-	-	-
Aortic rupture - not measured	-	-	-	-	-
Aneurysm growth rate - not measured	-	-	-	-	-
Number of patients with at least one cardiovascular complication - not measured	-	-	-	-	-
Number of patients who reached threshold for surgery - not measured	-	-	-	-	-

* The risk in the exercise group (and its 95% confidence interval) is based on the assumed risk in the comparison group and the relative effect of the intervention (and its 95% CI); CI: Confidence interval. GRADE Working Group grades of evidence. High certainty: We are very confident that the true effect lies close to that of the estimate of the effect. Moderate certainty: We are moderately confident in the effect estimate: The true effect is likely to be close to the estimate of the effect, but there is a possibility that it is substantially different. Low certainty: Our confidence in the effect estimate is limited: The true effect may be substantially different from the estimate of the effect. Very low certainty: We have very little confidence in the effect estimate: The true effect is likely to be substantially different from the estimate of effect.

**Table 6:** GRADEpro-GDT judgment of the quality of the evidence: GRADE question: Should exercise be indicated for patients with aortic aneurysms before and after surgery?

**Summary of findings:**						
**Exercises compared to no exercise for aortic aneurysm patients before and after surgery**						
**Patient or population: aortic aneurysm patients before and after surgery**						
**Setting:**						
**Intervention: exercise**						
**Comparison: no exercise**						
**Outcomes**	**Anticipated absolute effects** ^*^ **(95% CI)**		**Relative effect**	**Nº of participants**	**Certainty of the evidence**	**Comments**
	**Risk with usual care**	**Risk with physical exercises**	**(95% CI)**	**(studies)**	**(GRADE)**	
Mortality - not measured	-	-	-	-	-	
Aortic rupture - not measured	-	-	-	-	-	
Aneurysm growth rate - not measured	-	-	-	-	-	
Number of patients with at least one cardiovascular complication - not measured	-	-	-	-	-	
Number of patients who reached threshold for surgery - not measured	-	-	-	-	-	

* The risk in the exercise group (and its 95% confidence interval) is based on the assumed risk in the comparison group and the relative effect of the intervention (and its 95% CI).

CI: Confidence interval. GRADE Working Group grades of evidence. High certainty: We are very confident that the true effect lies close to that of the estimate of the effect. Moderate certainty: We are moderately confident in the effect estimate: The true effect is likely to be close to the estimate of the effect, but there is a possibility that it is substantially different. Low certainty: Our confidence in the effect estimate is limited: The true effect may be substantially different from the estimate of the effect. Very low certainty: We have very little confidence in the effect estimate: The true effect is likely to be substantially different from the estimate of effect.

## RESULTS

**Figure 1:**
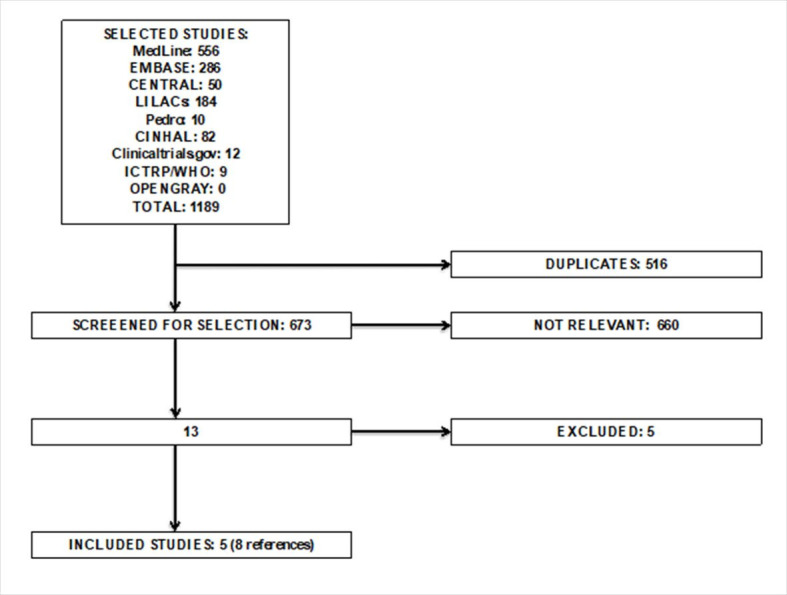
PRISMA flow chart for the review. This figure shows the PRISMA flow chart illustrating the processing of searching for, selecting, excluding, and including studies. There were three references from the same studies: two^25^^,^^26^ from Myers et al.^11^ and one^27^ from Barakat et al.,^28^ resulting in eight references from five original studies.

The search strategy returned 1189 references (Figure 1). From these, 8 references from 5 clinical trials involving a total of 387 participants were included (212 participants in exercise groups and 175 in no exercise groups).^11^^,^^18^^,^^28^^-^30 Three studies were conducted during the surveillance period.^11^^,^^18^^,^^29^ One study was conducted in the preoperative period (preoperative study).^28^ Finally, one study was performed in both the preoperative and postoperative periods (preoperative and postoperative study).^30^ The clinical trial authors also published three other articles based on the same studies: two^25^^,^^26^ from Myers et al.^11^ and one^27^ from Barakat et al.^28^

Table 7 describes the studies and their characteristics, including the reasons for inclusion or exclusion.

**Table 7:** Studies and characteristics.

**Included studies:**	**Characteristics:**
Kothmann et al. (2009)^29^	Number of patients in exercise group: 20
	Number of patients in control group: 10
	Age (mean): exercise group: 69.5 years, control group: 69.4 years
	Time of intervention: At surveillance
	Sex: 20 men and 5 women
	Interventions: ^#^ *“Exercise sessions of 30 min exercise on static Life Fitness bicycle with a 5 min warm-up and cooling down period. Participants attended twice weekly in groups of 3 to 4 patients.*
	*Participants were required to exercise in zones 12 to 14 on the Borg scale.”* 29
	Outcomes: change in anaerobic threshold
	Follow-up: 7 weeks
Tew et al. (2012)^18^	Number of patients in exercise group: 14
	Number of patients in control group: 14
	Age: exercise group: 71 ± 8 years, control group: 74 ± 6 years.
	Time of intervention: at surveillance
	Sex: male/female: Exercise group: 10/1. Control group: 11/3
	Interventions: ^**^ *“monitored physical exercises three times a week for 35 to 45 minutes at clinical unit; static Life Fitness bicycle in groups of 3 to 4 patients; treadmill walking; to expend up to 2000 Kcal.wk^-1^.”*
	Borg perceived exhaustion scale at zones of 12 to 14^18^.
	Outcomes: **“anaerobic threshold; quality of life; safety; and blood markers (including C reactive protein, matrix metalloproteinase-9, and glycemia)”
	Follow-up: 12 weeks
Myers et al. (2014)^11^	Number of patients in exercise group: 72
	Number of patients in control group: 68
	Age: exercise group: 71.8 ± 7 years, control group: 71.3 ± 8 years.
	Time of intervention: at surveillance
	Sex: exercise group: 92% men, control group: 93% men.
	Interventions: ^††^ *“monitored physical exercises three times a week workout for 45 minutes at clinical unit or home or both locations; including static Life Fitness bicycle in groups of 3 to 4 patients; treadmill walking; stair climbing; elliptical training; rowing; to expend up to 2000 Kcal.wk^-1^.”*
	Borg perceived exhaustion scale at zones of 12 to 14^11^.
	Outcomes: safety; aneurysm growth rates.
	Follow-up: up to 36 months
Barakat et al. (2016)^28^	Number of patients in exercise group: 62
	Number of patients in control group: 62
	Age: exercise group: 73.8 years, control group: 72.9 years.
	Time of intervention: during preoperative period.
	Sex: exercise group: 6 women; control group: 7 women.
	Interventions: ^‡‡^ “*5-minute warm up and stretching, cycle ergometer against moderate resistance for 2 minutes, heel-raise repetitions for 2 minutes, knee extensions against resistance repetitions for 2 minutes, dumbbells’ biceps/arm curls repetitions for 2 minutes, step-up lunges repetitions for 2 minutes, knee bends (bodyweight) repetitions for 2 minutes, and 5 minutes for cool down and stretching. Between each of the exercise stations, patients either walked around the gym or on a treadmill or rested for 2 minutes before moving on to the next exercise.”*
	Outcomes: composite postoperative cardiac, renal and postoperative respiratory complications; length of hospital stay, and ITU stay, “*APACHE II scores recorded at HDU/ITU admission, the occurrence of systemic inflammatory response syndrome (SIRS), 30-day mortality, postoperative bleeding requiring reoperation or transfusion of more than 4 units of blood products within 72 hours, and the need for reoperation.”*
	Follow-up: 12 weeks
Wnuk et al. (2016)^30^	Number of patients in exercise group: 44 (22 backward walking; 22 forward walking)
	Number of patients in control group: 21
	Age: exercise group: 71 ± 8 years, control group: 74 ± 6 years.
	Time of intervention: during postoperative period.
	Sex ratio: male/female: Exercise group: 10/1. Control group: 11/3.
	Interventions: postoperative backward walking training, and postoperative forward walking training.
	Outcomes: six minutes walking test; heart rate training; standard metabolic equivalent; FVC, FEV1, FEV1/FVC, PEF, hospital-stay.
	Follow-up: 7 days
**Excluded studies:**	**Reasons for exclusion:**
Dronkers et al. (2008)^31^	RCT comparing respiratory physical therapy with usual care in patients with an aortic aneurysm scheduled for surgery. Physical therapy was not considered to be exercise.
Nakayama et al. (2018)^32^	Retrospective cohort comparing cardiac rehabilitation with usual care with a follow up of 3000 days. Not a clinical trial.
	Main ID: JPRN-UMIN000028237
Hayashi et al. (2016)^33^	Case-control study. Patients were allocated to fit or unfit groups according to physical capacity.
Bailey et al. (2018)^34^	RCT evaluating effect of acute exercise on endothelial function in patients with abdominal aortic aneurysm. The study describes acute flow-mediated-dilatation and not the outcomes of the exercise over a long period as a necessity of treatment for the disease, so it was considered physical activity and not physical exercises.
Weston et al. (2017)^35^	RCT assessing the accuracy of high-intensity interval training (HIT) in patients awaiting repair of large abdominal aortic aneurysms.
**Ongoing studies:**	**Characteristics:**
ClinicalTrials.gov (2017)^36^	As contacted by e-mail: NCT01805973 (14) has been changed to “The AAA Get fit trial”:
	Randomized, parallel, blinded to assessors study: ^*^ *“…to explore the effectiveness of a 20-week community (either home or gym-based) exercise programme to achieve sustained improvements in peak VO2 and AT, as measured by CPET, in AAA patients. Changes in QoL, habitual activity levels and cardiovascular risk will also be assessed.”*
	ClinicalTrials.gov Identifier: NCT02997618
ClinicalTrials.gov (2017)^37^	Randomized, parallel, open study: ^†^ *“…to establish if it is possible for patients who have undergone major body surgery to complete a home based exercise training program and complete the assessments required to measure physical and cognitive function … [and] ... whether it is possible to improve the physical function of older patients undergoing major abdominal surgery in the period following surgery by using a simple exercise regimen that can be carried out at home..”*
	ClinicalTrials.gov Identifier: NCT03064308
ClinicalTrials.gov (2017)^38^	Randomized, parallel, blinded to assessors study: ^‡^ *“…comparing the effect of a “prehabilitation” program to usual care on quality of life and clinical outcomes in patients undergoing elective repair of their thoracic aorta.”*
	ClinicalTrials.gov Identifier: NCT02767518
Mosk 2017^39^	Non-randomized, two or more arms study:
	|| *“to evaluate if Multicomponent prehabilitation will reduce postoperative adverse events, primary delirium, which will result in less long-term adverse consequences.”*
	Main ID: NTR5932

Excluded studies: describes excluded studies and reasons for exclusion. Ongoing studies: describes ongoing studies and characteristics. Included studies: describes included studies and characteristics. AAA: Abdominal Aortic Aneurysm. AT: Anaerobic Threshold. APACHE II scores: Acute Physiology And Chronic Health Evaluation II. CEPET: Cardiopulmonary Exercise Test. FEV1: Forced Expiratory Volume in 1 second. FEV1/FVC: ratio of forced expiratory volume in one second to forced vital capacity. FVC: Functional Vital Capacity. HDU/ITU: High-dependency Unit/Intensive Care Unit. HIT: High-Intensity Interval Training. ITU: Intensive Care Unit. PEF: Peak Expiratory Flow. QoL: Quality of Life. RCT: Randomized Controlled Trial. VO2: rate of oxygen consumption during incremental exercise.

* Text quoted from ClinicalTrials.gov^36^;

† Text quoted from ClinicalTrials.gov^37^;

‡ Text quoted from ClinicalTrials.gov^38^;

^||^ Text quoted from Mosk^39^;

# Text quoted from Kothmann et al.^29^;

** Text quoted from Tew et al.^18^;

†† Text quoted from Myers et al.^11^;

‡‡ Text quoted from Barakat et al.^28^.

### Risk of bias in included studies

The Cochrane risk of bias table was used as follows:

Random sequence generation (selection bias): Kothmann et al.,^29^ Barakat et al.,^28^ and Wnuk et al.^30^ described their randomization methods and were classified as “low risk.” The remaining studies did not describe their randomization methods and were classified as “unclear risk.”^11^^,^^18^

Allocation concealment (performance bias and detection bias): all studies were judged to have an “unclear risk” of bias because the allocation method was not described.^11^^,^^18^^,^^28^^-^30

Blinding of personnel and participants (performance bias): Due to the nature of the intervention, it was presumably impossible to blind participants and personnel. Therefore, all studies were classified as “high risk” for this domain.

Blinding of outcome assessment (detection bias): Myers et al.^11^ described blinding of the outcome assessment, but the assessors who performed the blinding were not described. Tew et al.^18^ described their study as an open study. Thus, these two studies were judged as “unclear risk.” Kothmann et al.^29^ and Wnuk et al.^30^ responded by email regarding the blinding of the outcome assessment and were judged as “low risk.” Barakat et al.^28^ described blinding of the outcome assessment and was also judged as “low risk.”

Incomplete outcome data (attrition bias): Kothmann et al.,^29^ Tew et al.,^18^ and Wnuk et al.^30^ described >20.00% to 27.41% of losses to follow-up and the reasons for these losses. How this could impact the results was not clear; therefore, they were graded as “unclear risk.” Myers et al.^11^ was graded as “high risk” because the reasons for the 54% loss to follow-up were uncertain. Barakat et al.^28^ described no loss to follow-up for the proposed outcomes, and their study was judged “low risk.”

Selective reporting (reporting bias): All studies described every proposed outcome and were therefore considered to have a “low risk” of bias.

Other potential sources of bias: Myers et al.^11^ described a baseline imbalance between the groups with respect to body mass index (p = 0.002) and the prevalence of diabetes (30% in the exercise group vs. 12% in the usual care group [p = 0.01]). To what extent these imbalances could affect the results remained unclear. Tew et al.^18^ and Kothmann et al.^29^ did not describe the balance between the intervention and control groups because their study included no p values. These studies were classified as “unclear risk.” Barakat et al.^28^ and Wnuk et al.^30^ reported no baseline imbalances. Barakat et al.^28^ used two interventions with different prognoses (endovascular and open surgery), but the number of interventions was balanced between the groups. Therefore, their study was judged “low risk.” There was no other source of bias detected in the study by Wnuk et al.^30^; therefore, the study was also judged “low risk.”

The authors were contacted, and Kothmann et al.^29^ replied that it is “*totally inappropriate to conduct significance tests on baseline values,”* citing Senn.^40^ Additionally, these authors did not provide a significance test for the baseline values. This systematic review follows the Cochrane Handbook for Systematic Reviews of Interventions, which recommends inclusion of imbalances between groups in the domain “other source of bias.”^19^ The reasons for each judgment are presented in Table 8.

**Table 8:** Risk of bias table with justifications.

A) Clinical trials:	
**Study/bias**	**Support for judgment**
Barakat et al. 28	Quote: “*Randomization was performed using opaque, sealed, identical envelopes containing the treatment allocation, according to a computer-generated sequence prepared by an independent professional. Patients were randomized into one of the 2 groups—the exercise (intervention) group or the standard treatment (control) group. The randomization process was witnessed by an independent research professional and was carried out during the initial visit after obtaining informed consent, but before preoperative assessments and interventions*.”^28^ (p. 48).
Random sequence generation (selection bias)	
“Low risk”	Comment: randomization was described and seems to be appropriate.
Allocation concealment (selection bias) “Unclear risk”	Comment: not described
Blinding of participants and personnel (performance bias) – all-cause mortality	Quote: “*Clinicians including consultant surgeons, anesthetists, department’s medical and nursing staff, and interventional radiologists were blinded to patient group allocation. This was ensured by explaining the importance of blinding to all study participants and performing all study procedures in the separate Academic department*.”^28^ (p. 48).
“High risk”	Comment: due to the nature of the intervention, it is impossible to blind patients.
Blinding of participants and personnel (performance bias) – number of patients with aortic rupture.	Quote: “*Clinicians including consultant surgeons, anesthetists, department’s medical and nursing staff, and interventional radiologists were blinded to patient group allocation. This was ensured by explaining the importance of blinding to all study participants and performing all study procedures in the separate Academic department*.”^28^ (p. 48).
“High risk”	Comment: due to the nature of the intervention it is impossible to blind patients.
Blinding of participants and personnel (performance bias) – aneurysm growth	Quote: “*Clinicians including consultant surgeons, anesthetists, department’s medical and nursing staff, and interventional radiologists were blinded to patient group allocation. This was ensured by explaining the importance of blinding to all study participants and performing all study procedures in the separate Academic department*.”^28^ (p. 48).
“High risk”	Comment: due to the nature of the intervention it is impossible to blind patients.
Blinding of participants and personnel (performance bias) – quality of life	Not assessed
Blinding of participants and personnel (performance bias) – number of patients referred for surgery	Not assessed
Blinding of participants and personnel (performance bias) – peri-operative complications	Quote: “*Clinicians including consultant surgeons, anesthetists, department’s medical and nursing staff, and interventional radiologists were blinded to patient group allocation. This was ensured by explaining the importance of blinding to all study participants and performing all study procedures in the separate Academic department*.”^28^ (p. 48).
“High risk”	Comment: due to the nature of the intervention it is impossible to blind patients.
Há Blinding of participants and personnel (performance bias) – postoperative complications	Quote: “*Clinicians including consultant surgeons, anesthetists, department’s medical and nursing staff, and interventional radiologists were blinded to patient group allocation. This was ensured by explaining the importance of blinding to all study participants and performing all study procedures in the separate Academic department.*”^28^ (p. 48).
“High risk”	Comment: due to the nature of the intervention it is impossible to blind patients.
Blinding of participants and personnel (performance bias) – cardiovascular mortality	Quote: “*Clinicians including consultant surgeons, anesthetists, department’s medical and nursing staff, and interventional radiologists were blinded to patient group allocation. This was ensured by explaining the importance of blinding to all study participants and performing all study procedures in the separate Academic department.”* 28 (p. 48).
“High risk”	Comment: due to the nature of the intervention it is impossible to blind patients.
Blinding of participants and personnel (performance bias) – hospital stay	Quote: “*Clinicians including consultant surgeons, anesthetists, department’s medical and nursing staff, and interventional radiologists were blinded to patient group allocation. This was ensured by explaining the importance of blinding to all study participants and performing all study procedures in the separate Academic department*.”^28^ (p. 48).
“High risk”	Comment: due to the nature of the intervention it is impossible to blind patients.
Blinding of participants and personnel (performance bias) – VEF1	Not assessed
Blinding of outcome assessment (detection bias) – all-cause mortality	Quote: “*Clinicians including consultant surgeons, anesthetists, department’s medical and nursing staff, and interventional radiologists were blinded to patient group allocation. This was ensured by explaining the importance of blinding to all study participants and performing all study procedures in the separate Academic department.”* 28 (p. 48).
“Low risk”	Comment: blinding of outcome assessment was described and seems to be appropriate
Blinding of outcome assessment (detection bias) – number of patients with aortic rupture	Quote: “*Clinicians including consultant surgeons, anesthetists, department’s medical and nursing staff, and interventional radiologists were blinded to patient group allocation. This was ensured by explaining the importance of blinding to all study participants and performing all study procedures in the separate Academic department*.”^28^ (p. 48).
“Low risk”	Comment: blinding of outcome assessment was described and seems to be appropriate
Blinding of outcome assessment (detection bias) – aneurysm growth	Quote: “*Clinicians including consultant surgeons, anesthetists, department’s medical and nursing staff, and interventional radiologists were blinded to patient group allocation. This was ensured by explaining the importance of blinding to all study participants and performing all study procedures in the separate Academic department.*”^28^ (p. 48).
“Low risk”	Comment: blinding of outcome assessment was described and seems to be appropriate
Blinding of outcome assessment (detection bias) – quality of life	Not assessed
Blinding of outcome assessment (detection bias) – number of patients referred for surgery	Not applicable
Blinding of outcome assessment (detection bias) – peri-operative complications	Quote: “*Clinicians including consultant surgeons, anesthetists, department’s medical and nursing staff, and interventional radiologists were blinded to patient group allocation. This was ensured by explaining the importance of blinding to all study participants and performing all study procedures in the separate Academic department.*”^28^ (p. 48).
“Low risk”	Comment: blinding of outcome assessment was described and seems to be appropriate
Blinding of outcome assessment (detection bias) – postoperative complications	Quote: “*Clinicians including consultant surgeons, anesthetists, department’s medical and nursing staff, and interventional radiologists were blinded to patient group allocation. This was ensured by explaining the importance of blinding to all study participants and performing all study procedures in the separate Academic department.”* 28 (p. 48).
“Low risk”	Comment: blinding of outcome assessment was described and seems to be appropriate
Blinding of outcome assessment (detection bias) – cardiovascular mortality	Quote: “*Clinicians including consultant surgeons, anesthetists, department’s medical and nursing staff, and interventional radiologists were blinded to patient group allocation. This was ensured by explaining the importance of blinding to all study participants and performing all study procedures in the separate Academic department.”* 28 (p. 48).
“Low risk”	Comment: blinding of outcome assessment was described and seems to be appropriate
Blinding of outcome assessment (detection bias) – hospital stay	Quote: “*Clinicians including consultant surgeons, anesthetists, department’s medical and nursing staff, and interventional radiologists were blinded to patient group allocation. This was ensured by explaining the importance of blinding to all study participants and performing all study procedures in the separate Academic department.”* 28 (p. 48).
“Low risk”	Comment: blinding of outcome assessment was described and seems to be appropriate
Blinding of outcome assessment (detection bias) – VEF1	Not assessed
Incomplete outcome data (attrition bias) – all-cause mortality	Quote: “*Twelve patients—6 from each group—withdrew from the study before operative interventions as their procedures were cancelled or postponed. No patients were lost to follow-up. Sixty-two patients from each group were included in the final analysis.*”^28^
“Low risk”	Comment: there were 8.8% losses from each group and they were explained. There were no other loses of follow up.
Incomplete outcome data (attrition bias) – number of patients with aortic rupture	Quote: “*Twelve patients—6 from each group—withdrew from the study before operative interventions as their procedures were cancelled or postponed. No patients were lost to follow-up. Sixty-two patients from each group were included in the final analysis.*”^28^
“Low risk”	Comment: there were 8.8% losses from each group and they were explained There were no other loses of follow up.
Incomplete outcome data (attrition bias) – aneurysm growth	Quote: “*Twelve patients—6 from each group—withdrew from the study before operative interventions as their procedures were cancelled or postponed. No patients were lost to follow-up. Sixty-two patients from each group were included in the final analysis.*” 28
“Low risk”	Comment: there were 8.8% losses from each group and they were explained There were no other loses of follow up.
Incomplete outcome data (attrition bias) – quality of life	Not assessed
Incomplete outcome data (attrition bias) – number of patients referred for surgery	Not applicable
Incomplete outcome data (attrition bias) – peri-operative complications	Quote: “*Twelve patients—6 from each group—withdrew from the study before operative interventions as their procedures were cancelled or postponed. No patients were lost to follow-up. Sixty-two patients from each group were included in the final analysis.*” 28
“Low risk”	Comment: there were 8.8% losses from each group and they were explained There were no other loses of follow up.
Incomplete outcome data (attrition bias) – postoperative complications	Quote: “*Twelve patients—6 from each group—withdrew from the study before operative interventions as their procedures were cancelled or postponed. No patients were lost to follow-up. Sixty-two patients from each group were included in the final analysis.*” 28
“Low risk”	Comment: there were 8.8% losses from each group and they were explained There were no other loses of follow up.
Incomplete outcome data (attrition bias) – cardiovascular mortality	Quote: “*Twelve patients—6 from each group—withdrew from the study before operative interventions as their procedures were cancelled or postponed. No patients were lost to follow-up. Sixty-two patients from each group were included in the final analysis.*” 28
“Low risk”	Comment: there were 8.8% losses from each group and they were explained There were no other loses of follow up.
Incomplete outcome data (attrition bias) – hospital stay	Quote: “*Twelve patients—6 from each group—withdrew from the study before operative interventions as their procedures were cancelled or postponed. No patients were lost to follow-up. Sixty-two patients from each group were included in the final analysis.*” 28
“Low risk”	Comment: there were 8.8% losses from each group and they were explained There were no other loses of follow up.
Incomplete outcome data (attrition bias) – VEF1	Not assessed
Selective reporting (reporting bias)	Comment: A protocol has been published: NCT01062594. All proposed outcomes were reported.
“Low risk”	Comment: there were no imbalances between groups. There were two different interventions (endovascular and open surgery). The numbers were balanced between groups.
Other bias	
“Low risk”	
Kothmann et al.^29^	
Random sequence generation (selection bias)	Quote: *“...participants were randomly allocated (via sealed envelopes) to a supervised exercise intervention or to the control group (usual care).”* 29
“Low risk”	Comment: Randomization was described and seems to be appropriate.
Allocation concealment (selection bias)	Comment: not described
“Unclear risk”	
Blinding of participants and personnel (performance bias) – all-cause mortality	Comment: no information provided. Probably not done due to the nature of the intervention.
“High risk”	
Blinding of participants and personnel (performance bias) – number of patients with aortic rupture.	Comment: no information provided. Probably not done due to the nature of the intervention.
“High risk”	
Blinding of participants and personnel (performance bias) – aneurysm growth	Not assessed
Blinding of participants and personnel (performance bias) – quality of life	Not assessed
Blinding of participants and personnel (performance bias) – number of patients referred for surgery	Comment: no information provided. Probably not done due to the nature of the intervention.
“High risk”	
Blinding of participants and personnel (performance bias) – peri-operative complications	Not applicable
Blinding of participants and personnel (performance bias) – postoperative complications	Not applicable
Blinding of participants and personnel (performance bias) – cardiovascular mortality	Comment: no information provided. Probably not done due to the nature of the intervention.
“High risk”	
Blinding of participants and personnel (performance bias) – hospital stay	Not applicable
Blinding of participants and personnel (performance bias) – VEF1	Not assessed
Blinding of outcome assessment (detection bias) – all-cause mortality	Quote: *“In the paper we state: “The investigator reading AT results (G.D.) was blinded to group allocation.” Simply, GD was provided with the output from the cardiopulmonary exercise tests and derived the anaerobic threshold for each participant without knowledge of group assignment, i.e., blind.”* 29 Provided by email
“Low risk”	Comment: blinding of outcome assessment properly described.
Blinding of outcome assessment (detection bias) – number of patients with aortic rupture	Quote: *“In the paper we state: “The investigator reading AT results (G.D.) was blinded to group allocation.” Simply, GD was provided with the output from the cardiopulmonary exercise tests and derived the anaerobic threshold for each participant without knowledge of group assignment, i.e., blind.”* 29 Provided by email
“Low risk”	Comment: blinding of outcome assessment properly described.
Blinding of outcome assessment (detection bias) – aneurysm growth	Not assessed
Blinding of outcome assessment (detection bias) – quality of life	Not assessed
Blinding of outcome assessment (detection bias) – number of patients referred for surgery	Quote: *“In the paper we state: “The investigator reading AT results (G.D.) was blinded to group allocation.” Simply, GD was provided with the output from the cardiopulmonary exercise tests and derived the anaerobic threshold for each participant without knowledge of group assignment, i.e., blind.”* 29 Provided by email
“Low risk”	Comment: blinding of outcome assessment properly described.
Blinding of outcome assessment (detection bias) – peri-operative complications	Not applicable
Blinding of outcome assessment (detection bias) – postoperative complications	Not applicable
Blinding of outcome assessment (detection bias) – cardiovascular mortality	Quote: *“In the paper we state: “The investigator reading AT results (G.D.) was blinded to group allocation.” Simply, GD was provided with the output from the cardiopulmonary exercise tests and derived the anaerobic threshold for each participant without knowledge of group assignment, i.e., blind.”* 29 Provided by email
“Low risk”	Comment: blinding of outcome assessment properly described.
Blinding of outcome assessment (detection bias) – hospital stay	Not applicable
Blinding of outcome assessment (detection bias) – VEF1	Not assessed
Incomplete outcome data (attrition bias) – all-cause mortality	“*Of these, 17 of 20 and eight of 10 completed the study period in the exercise and control groups, respectively, producing full data sets for analysis*”^29^
“Unclear risk”	Comment: (There was 15% losses from the intervention group and 20% from the control group. We are not sure about the extent to which this could affect the results).
Incomplete outcome data (attrition bias) – number of patients with aortic rupture	“*Of these, 17 of 20 and eight of 10 completed the study period in the exercise and control groups, respectively, producing full data sets for analysis*”^29^
“Unclear risk”	Comment: (There was 15% losses from the intervention group and 20% from the control group. We are not sure about the extent to which this could affect the results).
Incomplete outcome data (attrition bias) – aneurysm growth	Not assessed
Incomplete outcome data (attrition bias) – quality of life	Not assessed
Incomplete outcome data (attrition bias) – number of patients referred for surgery	“*Of these, 17 of 20 and eight of 10 completed the study period in the exercise and control groups, respectively, producing full data sets for analysis*”^29^
“unclear risk”	Comment: There was 15% losses from the intervention group and 20% from the control group. We are not sure to what extent this could affect the results.
Incomplete outcome data (attrition bias) – peri-operative complications	Not applicable
Incomplete outcome data (attrition bias) – postoperative complications	Not applicable
Incomplete outcome data (attrition bias) – cardiovascular mortality	“*Of these, 17 of 20 and eight of 10 completed the study period in the exercise and control groups, respectively, producing full data sets for analysis*”^29^
“Unclear risk”	Comment: There was 15% losses from the intervention group and 20% from the control group. We are not sure to what extent this could affect the results.
Incomplete outcome data (attrition bias) – hospital stay	Not applicable
Incomplete outcome data (attrition bias) – VEF1	Not assessed
Selective reporting (reporting bias)	Comment: All the proposed outcomes were reported.
“Low risk”	
Other bias	Comment: There is an uncertainty about the balance between groups at baseline, since no p value was provided. We are not sure to what extent this could affect the results.
“Unclear risk”	
Myers et al.^11^	
Random sequence generation (selection bias)	Quote: “*One hundred and forty patients with small AAAs (72 T 8 yr) were randomised to exercise training (n = 72) or usual care (n = 68)”* 11
“Unclear risk”	Comment: Not described.
Allocation concealment (selection bias)	Comment: not described
“Unclear risk”	
Blinding of participants and personnel (performance bias) – all-cause mortality	Comment: not stated. Probably not done since the nature of intervention precluded this masking
“High risk”	
Blinding of participants and personnel (performance bias) – number of patients with aortic rupture.	Comment: not stated. Probably not done since the nature of intervention precluded this masking
“High risk”	
Blinding of participants and personnel (performance bias) – aneurysm growth	Comment: not stated. Probably not done since the nature of intervention precluded this masking
“High risk”	
Blinding of participants and personnel (performance bias) – quality of life	Not assessed
Blinding of participants and personnel (performance bias) – number of patients referred for surgery	Comment: not stated. Probably not done since the nature of intervention precluded this masking
“High risk”	
Blinding of participants and personnel (performance bias) – peri-operative complications	Not assessed
Blinding of participants and personnel (performance bias) – postoperative complications	Not assessed
Blinding of participants and personnel (performance bias) – cardiovascular mortality	Comment: not stated. Probably not done since the nature of intervention precluded this masking
“High risk”	
Blinding of participants and personnel (performance bias) – hospital stay	Not assessed
Blinding of participants and personnel (performance bias) – VEF1	Not assessed
Blinding of outcome assessment (detection bias) – all-cause mortality	Quote: *“Both the RVT and the individual making the diameter measurements were blinded to group randomisation”. Answered by e-mail: “both were unaware of the intervention or control group.”* 11
“Unclear risk”	Comment: no information provided for data assessors
Blinding of outcome assessment (detection bias) – number of patients with aortic rupture	Quote: Quote: *“Both the RVT and the individual making the diameter measurements were blinded to group randomisation”. Answered by e-mail: “both were unaware of the intervention or control group.”* 11
“Unclear risk”	Comment: no information provided for data assessors
Blinding of outcome assessment (detection bias) – aneurysm growth	Quote: *“Both the RVT and the individual making the diameter measurements were blinded to group randomisation”. Answered by e-mail: “both were unaware of the intervention or control group.”* 11
“Unclear risk”	Comment: no information provided for data assessors
Blinding of outcome assessment (detection bias) – quality of life	Not assessed
Blinding of outcome assessment (detection bias) – number of patients referred for surgery	Quote: *“Both the RVT and the individual making the diameter measurements were blinded to group randomisation”. Answered by e-mail: “both were unaware of the intervention or control group.”* 11
“Unclear risk”	Comment: no information provided for data assessors
Blinding of outcome assessment (detection bias) – peri-operative complications	Not assessed
Blinding of outcome assessment (detection bias) – postoperative complications	Not assessed
Blinding of outcome assessment (detection bias) – cardiovascular mortality	Quote: *“Both the RVT and the individual making the diameter measurements were blinded to group randomisation”. Answered by e-mail: “both were unaware of the intervention or control group.”* 11
“Unclear risk”	Comment: no information provided for data assessors
Blinding of outcome assessment (detection bias) – hospital stay	Not assessed
Blinding of outcome assessment (detection bias) – VEF1	Not assessed
Incomplete outcome data (attrition bias) – all-cause mortality	Quote: *“81% of subjects completed at least 1 year in the trial”* 11
“High risk”	Comment: There were 19% losses in one year. Additionally, at the end of follow-up, there were 39 losses from the intervention group and 36 from the control group. The reasons for these losses are unclear.
Incomplete outcome data (attrition bias) – number of patients with aortic rupture	Quote: *“81% of subjects completed at least 1 year in the trial”* 11
“High risk”	Comment: There were 19% losses in one year. Additionally, at the end of follow-up, there were 39 losses from the intervention group and 36 from the control group. The reasons for these losses are unclear.
Incomplete outcome data (attrition bias) – aneurysm growth	Quote: *“81% of subjects completed at least 1 year in the trial”* 11
“High risk”	Comment: There were 19% losses in one year. Additionally, at the end of follow-up, there were 39 losses from the intervention group and 36 from the control group. The reasons for these losses are unclear.
Incomplete outcome data (attrition bias) – quality of life	Not assessed
Incomplete outcome data (attrition bias) – number of patients referred for surgery	Quote: *“81% of subjects completed at least 1 year in the trial”* 11
“High risk”	Comment: There were 19% losses in one year. Additionally, at the end of follow-up, there were 39 losses from the intervention group and 36 from the control group. The reasons for these losses are unclear.
Incomplete outcome data (attrition bias) – peri-operative complications	Not assessed
Incomplete outcome data (attrition bias) – postoperative complications	Not assessed
Incomplete outcome data (attrition bias) – cardiovascular mortality	Quote: *“81% of subjects completed at least 1 year in the trial”* 11
“High risk”	Comment: There were 19% losses in one year. Additionally, at the end of follow-up, there were 39 losses from the intervention group and 36 from the control group. The reasons for these losses are unclear.
Incomplete outcome data (attrition bias) – hospital stay	Not assessed
Incomplete outcome data (attrition bias) – VEF1	Not assessed
Selective reporting (reporting bias)	Quote: Protocol available at Clinicaltrials.gov, identifier: NCT00349947.
“Low risk”	Comment: protocol described. All proposed outcomes were reported
Other bias	Comment: There was an “*imbalance between groups at baseline regarding BMI mean at baseline (p =0.002) and frequency of diabetes (30% vs. 12% in the exercise and usual care groups, respectively, P = 0.01)*.” 11
“Unclear risk”	We are not sure to what extent this could affect the results.
Tew et al.^18^	
Random sequence generation (selection bias)	Quote: *“Allocation to exercise or control was done using a randomization sequence created by an independent researcher before study commencement.”* 18
“Unclear risk”	Comment: unclear information
Allocation concealment (selection bias)	Quote: *“The study researchers were made aware of this sequence on a case-by-case basis after baseline assessments were completed.”* 18
“Unclear risk”	Comment: unclear information
Blinding of participants and personnel (performance bias) – all-cause mortality	Quote: *“Ventilatory threshold was determined by an independent exercise physiologist blinded to group allocation using the v-slope and ventilatory equivalents methods.”* 18
“High risk”	Comment: The nature of the intervention precluded this masking
Blinding of participants and personnel (performance bias) – number of patients with aortic rupture.	Quote: *“Ventilatory threshold was determined by an independent exercise physiologist blinded to group allocation using the v-slope and ventilatory equivalents methods.”* 18
“High risk”	Comment: The nature of the intervention precluded this masking
Blinding of participants and personnel (performance bias) – aneurysm growth	Quote: *“Ventilatory threshold was determined by an independent exercise physiologist blinded to group allocation using the v-slope and ventilatory equivalents methods.”* 18
“High risk”	Comment: The nature of the intervention precluded this masking
Blinding of participants and personnel (performance bias) – quality of life	Quote: *“Ventilatory threshold was determined by an independent exercise physiologist blinded to group allocation using the v-slope and ventilatory equivalents methods.”* 18
“High risk”	Comment: The nature of the intervention precluded this masking
Blinding of participants and personnel (performance bias) – number of patients referred for surgery	Not assessed
Blinding of participants and personnel (performance bias) – peri-operative complications	Not applicable
Blinding of participants and personnel (performance bias) – postoperative complications	Not applicable
Blinding of participants and personnel (performance bias) – cardiovascular mortality	Quote: *“Ventilatory threshold was determined by an independent exercise physiologist blinded to group allocation using the v-slope and ventilatory equivalents methods.”* 18
“High risk”	Comment: The nature of the intervention precluded this masking
Blinding of participants and personnel (performance bias) – hospital stay	Not applicable
Blinding of participants and personnel (performance bias) – VEF1	Not assessed
Blinding of outcome assessment (detection bias) – all-cause mortality	Quote: “*The study researchers were made aware of this sequence on a case-by-case basis after baseline assessments were completed”.* 18
“High risk”	Comment: There was no blinding
Blinding of outcome assessment (detection bias) – number of patients with aortic rupture	Quote: “*The study researchers were made aware of this sequence on a case-by-case basis after baseline assessments were completed”.* 18
“High risk”	Comment: There was no blinding
Blinding of outcome assessment (detection bias) – aneurysm growth	Quote: “*The study researchers were made aware of this sequence on a case-by-case basis after baseline assessments were completed”.* 18
“High risk”	Comment: There was no blinding
Blinding of outcome assessment (detection bias) – quality of life	Quote: *The study researchers were made aware of this sequence on a case-by-case basis after baseline assessments were completed”.* 18
“High risk”	Comment: There was no blinding
Blinding of outcome assessment (detection bias) – number of patients referred for surgery	Not assessed
Blinding of outcome assessment (detection bias) – peri-operative complications	Not applicable
Blinding of outcome assessment (detection bias) – postoperative complications	Not applicable
Blinding of outcome assessment (detection bias) – cardiovascular mortality	Quote: *The study researchers were made aware of this sequence on a case-by-case basis after baseline assessments were completed”.* 18
“High risk”	Comment: There was no blinding
Blinding of outcome assessment (detection bias) – hospital stay	Not applicable
Blinding of outcome assessment (detection bias) – VEF1	Not assessed
Incomplete outcome data (attrition bias) – all-cause mortality	Quote: *“Three participants did not complete the exercise intervention: 1 withdrew because of being diagnosed with cancer, 1 underwent pacemaker implantation, and 1 suffered a back injury at home”* 18
“Unclear risk”	Comment: There were about 20% of losses from the intervention group, with reasons provided. We are not sure to what extent this could affect the results.
Incomplete outcome data (attrition bias) – number of patients with aortic rupture	Quote: *“Three participants did not complete the exercise intervention: 1 withdrew because of being diagnosed with cancer, 1 underwent pacemaker implantation, and 1 suffered a back injury at home”* 18
“Unclear risk”	Comment: There were about 20% of losses from the intervention group, with reasons provided. We are not sure to what extent this could affect the results.
Incomplete outcome data (attrition bias) – aneurysm growth	Quote: *“Three participants did not complete the exercise intervention: 1 withdrew because of being diagnosed with cancer, 1 underwent pacemaker implantation, and 1 suffered a back injury at home”* 18
“Unclear risk”	Comment: There were about 20% of losses from the intervention group, with reasons provided. We are not sure to what extent this could affect the results.
Incomplete outcome data (attrition bias) – quality of life	Quote: *“Three participants did not complete the exercise intervention: 1 withdrew because of being diagnosed with cancer, 1 underwent pacemaker implantation, and 1 suffered a back injury at home”* 18
“Unclear risk”	Comment: There were about 20% of losses from the intervention group, with reasons provided. We are not sure to what extent this could affect the results.
Incomplete outcome data (attrition bias) – number of patients referred for surgery	Not assessed
Incomplete outcome data (attrition bias) – peri-operative complications	Not applicable
Incomplete outcome data (attrition bias) – postoperative complications	Not applicable
Incomplete outcome data (attrition bias) – cardiovascular mortality	Quote: *“Three participants did not complete the exercise intervention: 1 withdrew because of being diagnosed with cancer, 1 underwent pacemaker implantation, and 1 suffered a back injury at home”* 18
“Unclear risk”	Comment: There were about 20% of losses from the intervention group, with reasons provided. We are not sure to what extent this could affect the results.
Incomplete outcome data (attrition bias) – hospital stay	Not applicable
Incomplete outcome data (attrition bias) – VEF1	Not assessed
Selective reporting (reporting bias)	Quote: *“The study was registered in ClinicalTrials.gov under reference no. NCT01234610.”* 18
“Low risk”	Comment: there is a protocol and it seems to be appropriate. All proposed outcomes were reported.
	Does not show the data for the quality of life outcome**: *“...*** *or any of the 8 quality of life domains (P.05; data not presented)”* 18
Other bias	Comment: There is an uncertainty about the balance between groups at baseline, since no p value was provided. We are not sure to what extent this could affect the results.
“Unclear risk	
Wnuk et al.^30^	
Random sequence generation (selection bias)	Quote: *“The randomization of the study was conducted by drawing envelopes containing a number of the appropriate group – single blind study. Patients with the number 1 were qualified for the experimental group with backward walking training (group I), with number 2 for the experimental group with forward walking training (group II) and 3 for the control group.”* 30
“Low risk”	Comment: randomization considered done and apparently appropriate.
Allocation concealment (selection bias)	Comment: not stated
“Unclear risk”	
Blinding of participants and personnel (performance bias) – all-cause mortality	Not assessed
Blinding of participants and personnel (performance bias) – number of patients with aortic rupture.	Not applicable
Blinding of participants and personnel (performance bias) – aneurysm growth	Not applicable
Blinding of participants and personnel (performance bias) – quality of life	Not assessed
Blinding of participants and personnel (performance bias) – number of patients referred for surgery	Not applicable
Blinding of participants and personnel (performance bias) – peri-operative complications	Not applicable
Blinding of participants and personnel (performance bias) – postoperative complications	Not assessed
Blinding of participants and personnel (performance bias) – cardiovascular mortality	Not assessed
Blinding of participants and personnel (performance bias) – hospital stay	Quote: “*single blind study”* 30
“High risk”	Comment: not blinded
Blinding of participants and personnel	Quote: “*single blind study”* 30
(performance bias) – VEF1	Comment: not blinded
“High risk”	
Blinding of outcome assessment (detection bias) – all-cause mortality	Not assessed
Blinding of outcome assessment (detection bias) – number of patients with aortic rupture	Not applicable
Blinding of outcome assessment (detection bias) – aneurysm growth	Not applicable
Blinding of outcome assessment (detection bias) – quality of life	Not assessed
Blinding of outcome assessment (detection bias) – number of patients referred for surgery	Not applicable
Blinding of outcome assessment (detection bias) – peri-operative complications	Not applicable
Blinding of outcome assessment (detection bias) – postoperative complications	Not assessed
Blinding of outcome assessment (detection bias) – cardiovascular mortality	Not assessed
Blinding of outcome assessment (detection bias) – hospital stay	Quote: described as a single blinded study. Replied by e-mail 09/27/2017: *“Measurement of gait parameters and spirometry was evaluated by physiotherapist from the Department of Rehabilitation. Routine physiotherapy and training walking in three groups was conducted by physiotherapist from the department of General and Vascular Surgery.”* 30
“Low risk”	Comment: blinding of outcome assessment was described and seems to be appropriate.
Blinding of outcome assessment (detection bias) – VEF1	Quote: described as a single blinded study. Replied by e-mail 09/27/2017: *“Measurement of gait parameters and spirometry was evaluated by physiotherapist from the Department of Rehabilitation. Routine physiotherapy and training walking in three groups was conducted by physiotherapist from the department of General and Vascular Surgery.”* 30
“Low risk”	Comment: blinding of outcome assessment was described and seems to be appropriate.
Incomplete outcome data (attrition bias) – all-cause mortality	Not assessed
Incomplete outcome data (attrition bias) – number of patients with aortic rupture	Not applicable
Incomplete outcome data (attrition bias) – aneurysm growth	Not applicable
Incomplete outcome data (attrition bias) – quality of life	Not assessed
Incomplete outcome data (attrition bias) – number of patients referred for surgery	Not applicable
Incomplete outcome data (attrition bias) – peri-operative complications	Not applicable
Incomplete outcome data (attrition bias) – postoperative complications	Not assessed
Incomplete outcome data (attrition bias) – cardiovascular mortality	Not assessed
Incomplete outcome data (attrition bias) – hospital stay	Comment: there were 27.41% dropouts and we do not what the impact of this would be on results and conclusions.
“Unclear risk”	
Incomplete outcome data (attrition bias) – VEF1	Comment: there were 27.41% dropouts and we do not what the impact of this would be on results and conclusions.
“Unclear risk”	
Selective reporting (reporting bias)	Comment: the proposed outcomes were described and seem to be appropriate.
“Low risk”	
Other bias	Comment: describes balance between groups at baseline. No other source of bias detected.
“Low risk”	

Not assessed: The reduction in bias is possible since the authors report the necessary information to avoid bias but it was not described in the study; Not applicable: not possible within the study protocol; Not stated: not described.

### Effects of interventions

The following comparisons were analyzed:

Comparison 1: Exercise for patients with small aneurysms during surveillance.^11^^,^^18^^,^^29^

In total, 106 subjects in the exercise group underwent a 7-week^29^ to 36-month^11^ supervised exercise program, and 92 were included in no exercise groups.

**Figure 2:**
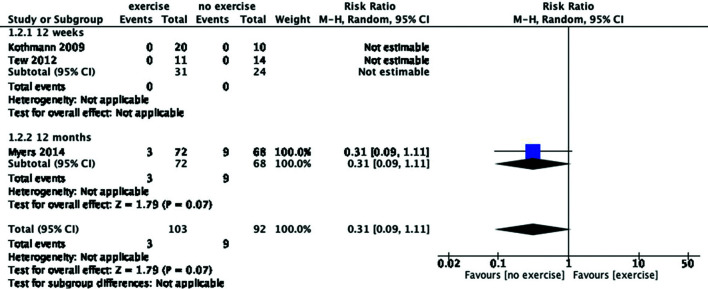
Number of patients referred for surgery at any time during surveillance. This figure shows patients referred for surgery at any time during the surveillance period. They were described at 12 weeks (1.2.1) and at 12 months (1.2.2). M-H, Mantel–Haenszel; Random, random-effects model; CI, confidence interval; Events, number of patients referred for surgery; Total, total number of patients; Total (95% CI), effect size at 95% confidence interval.
- Cardiovascular adverse events were not different between the intervention and control groups (RR, 1.57; 95% CI, 0.07–35.46).^11^^,^^18^^,^^29^ In the study by Kothmann et al.,^29^ one patient in the intervention group had a severe adverse cardiac event after seven sessions.

Proposed outcomes:

- No mortality was reported;
- No patients developed aneurysm rupture;
- The aneurysm growth rate did not change in the pooled studies from the 12-week to 12-month follow-up (mean difference [MD], −0.05; 95% confidence interval [CI], −0.13 to 0.03).^11^^,^^18^ Additionally, there was a statistical tendency to reach significance at the 95% CI;
- Despite the fact that Tew et al.^18^ described quality of life, no data were depicted. The study reported a non-significant change in eight evaluated domains;
- There was a tendency for the number of patients referred for surgery to reduce, but it was not statistically significant (risk ratio [RR], 0.31; 95% CI, 0.09–1.11) (Figure 2);^11^^,^^18^^,^^29^

**Figure 3:**
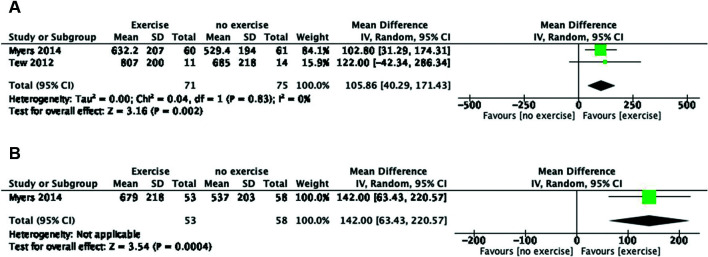
Exercise time during surveillance. (A) Exercise time during surveillance at 12 weeks (exercise vs. no exercise). (B) Exercise time during surveillance at 12 months (exercise vs. no exercise). IV, inverse variance; Random, random-effects model; CI, confidence interval; Total, total number of patients; Total (95% CI), effect size at 95% confidence interval.

Non-proposed outcomes:

There was an improvement in the exercise time at 12 weeks (MD, 105.86; 95% CI, 40.29–171.43) (Figure 3A).^11^^,^^18^ This result was even stronger in the 12-month clinical trial (MD, 142.00; 95% CI, 63.43–220.57) (Figure 3B).^11^

**Figure 4:**
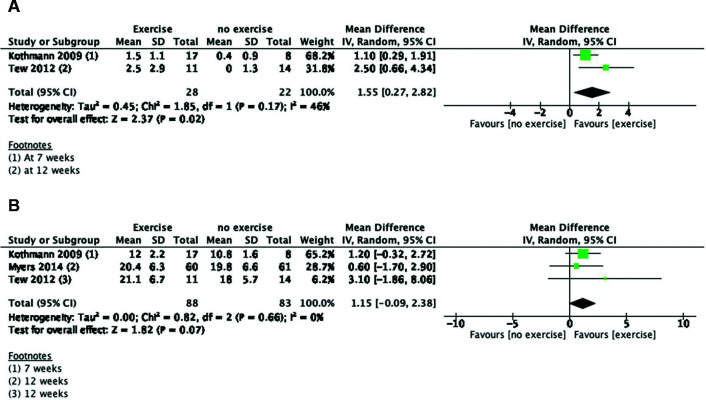
Change in anaerobic threshold and peak VO_2_ during surveillance. (A) Change in total anaerobic threshold values at 7 and 12 weeks during surveillance (exercise vs. no exercise). (B) Peak VO_2_ during surveillance at 7 and 12 weeks (exercise vs. no exercise). IV, inverse variance; Random, random-effects model; CI, confidence interval; Total, total number of patients; Total (95% CI), effect size at 95% confidence interval.

The change in anaerobic threshold improved after at least 7 weeks of exercise (MD, 1.55; 95% CI, 0.27–2.82) (Figure 4A).^11^^,^^18^^,^^29^ Although the maximal rate of oxygen consumption during incremental exercise (VO_2_ peak) improved, the difference did not attain statistical significance (MD, 1.15; 95% CI, −0.09 to 2.38) (Figure 4B).^11^^,^^18^^,^^29^

Comparison 2: Exercise in the preoperative period^28^

One study assessed this comparison.^28^ The study included 62 patients in the exercise group and 62 in the no exercise group for 6 weeks. Data were assessed in the interquartile range, which was transformed to standard deviation by dividing the interquartile range by 1.35, as described in the seventh chapter of the Cochrane handbook.^19^

**Figure 5:**
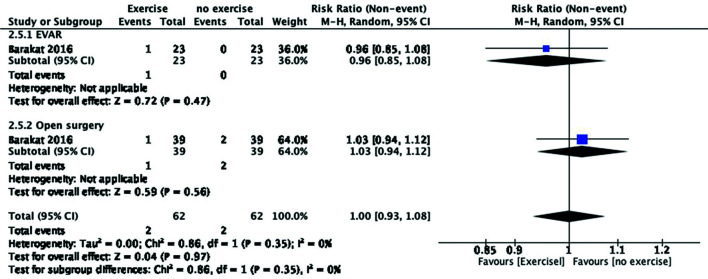
Thirty-day mortality after surgery in patients in the preoperative study. This figure compares 30-day mortality after surgery in patients in the exercise and no exercise groups during the preoperative period. EVAR, endovascular aneurysm repair. Comparison 2 involves the subset treated with EVAR (2.5.1) and the subset treated with open surgery (2.5.2). M-H: Mantel–Haenszel; Random, random-effects model; CI, confidence interval; Events, number of deaths up to 30 days after surgery; Total, total number of patients; Total (95% CI), effect size at 95% confidence interval.
- No participants developed an aneurysm rupture;
- Quality of life was not measured;
- Overall, postoperative complications were reduced in the exercise group (RR, 0.54; 95% CI, 0.31–0.93). In a subgroup analysis, cardiac complications (RR, 0.36; 95% CI, 0.14–0.93), and renal complications (RR, 0.31; 95% CI, 0.11–0.89) had the most important benefit. Despite a tendency to reduce pulmonary complications, this was not statistically significant (RR, 0.54; 95% CI, 0.23–1.26). When analyzed by surgical subgroups, renal complications were lower in open aneurysm surgery (RR, 0.54; 95% CI, 0.34–0.87) than in endovascular repair (RR, 1.00; 95% CI, 0.07–15.04). The same trend occurred in cardiac complications: open aneurysm repair (RR, 0.36; 95% CI, 0.13–1.04) versus an endovascular approach (RR, 0.33; 95% CI, 0.04–2.97). Pulmonary complications were not significantly reduced in endovascular repair (RR, 0.11; 95% CI, 0.01–1.95) or open repair (RR, 0.78; 95% CI, 0.32–1.88);
- Hospital stay was not reduced in endovascular repair (MD, −1.00; 95% CI, −4.22 to 2.22) or open aneurysm repair groups (MD, 0.00; 95% CI, −0.55 to 0.55);
- There was a detectable reduction in the critical care stay in the exercise group (MD, −1.00; 95% CI, −1.26 to −0.74).

Proposed outcomes:

- There was no difference in 30-day mortality between the groups (RR, 1.00; 95% CI, 0.93–1.08) (Figure 5);

Eleven of 62 patients who were referred for exercise (17.7%) did not attend the scheduled exercise sessions. There were no losses to follow-up after initiating the study.

Non-proposed outcomes:

Bleeding was described clinically or as a need for transfusion of more than four bags and was not affected by inclusion in either the preoperative exercise or no exercise study groups (RR, 0.57; 95% CI, 0.18–1.85).^28^

There was an improvement in anaerobic threshold (MD, 1.80; 95% CI, 0.68–2.92) and VO_2_ peak oxygen consumption (MD, 1.60; 95% CI, 0.40–2.80).^28^

Comparison 3: Exercise in the preoperative and postoperative periods^30^

One of the studies included assessed 22 patients who performed backward walking, 22 who performed forwarding walking, and 21 in a control group during the preoperative and postoperative periods. After contact, the author reported 18 drop-outs: 7 in the backward walking group (due to myocardial infarction in 3 patients, respiratory failure in 3, and refusal to exercise after surgery in 1), 6 in the forward walking group (myocardial infarction in 2 patients, respiratory failure in 2, and exclusion due to blood coagulation dysfunction in 2), and 5 patients in the control group (all due to myocardial infarction). A per-protocol analysis was conducted (including 15 patients in the backward walking group, 16 in the forwarding walking group, and 16 in the control group). All patients were men, and the results for proposed outcomes were as follows:

- No mortality was reported;
- Quality of life was not measured;
- The number of participants presenting with at least one severe complication was not reported;
- The hospital stay was detectably reduced in the forward walking group compared with the control group (MD, −0.69; 95% CI, −1.24 to −0.14). No difference was observed between the backward walking group and control group (MD, −0.06; 95% CI, −0.53 to 0.41);
- Length of intensive care unit stay after aneurysm surgery (in days) was not assessed;
- During forward walking, the forced expiratory volume in 1 second was not different between the intervention group and control group (RR, 0.27; 95% CI, −0.12 to 0.66).

The proposed subgroup analysis was not performed because of limited data available.

Using GRADEpro-GDT,^24^ we judged the quality of the evidence as “very low” for all outcomes. Quality ratings were downgraded due to methodological limitations (impossibility of blinding personnel and participants, attrition bias) and imprecision (single study for some outcomes and low numbers of participants) (Tables 2, 3, 4 and 5).

## DISCUSSION

This review revealed no differences in mortality rates between patients with and without exercise during surveillance, preoperative, or postoperative periods. Additionally, no aneurysm ruptures were detected in any intervention groups (total of 209 patients). These clinical trials did not identify reduction in aneurysmal expansion rates or referrals for surgery (Figure 2). However, 6 weeks of preoperative exercise was an effective intervention for reducing cardiac and renal complication rates after surgical interventions and also the length of critical care stay.^28^ Indeed, a forward walking program started before and continued after surgery reduced the hospital stay.^30^

A retrospective cohort with a longer follow-up showed that exercise is an effective intervention to reduce aneurysmal expansion and aortic aneurysm repair rates.^32^ This result is similar to that in an animal model study.^41^ Because these clinical trials had short-term follow-up and low numbers of patients, the effect direction may yet change with the addition of new studies.

Patients with aortic aneurysms have a life expectancy lower than that of individuals of the same age in the same population,^17^ and it has been recognized the exercise decreases mortality in patients with stable coronary heart disease.^42^ Although exercise did not reduce the mortality rates in this review, some mortality can be attributed to patients’ associated clinical risk factors.^17^ Exercise could also be advocated to improve patients’ quality of life, but data are insufficient to assess this outcome.^18^

With respect to safety concerns, in all studies exercise did not increase the risks of rupture, death, or severe cardiovascular adverse effects. Additionally, there are presumably large numbers of patients with undiagnosed small abdominal aortic aneurysms in exercise programs and rupture rates are low. Indeed, cardiorespiratory fitness is a marker of mortality,^43^ and improved fitness can be a valuable intervention to prevent at least serious complications whenever surgery is necessary. Good fitness levels are considered important to reduce hospital stay with no reduction in surgical mortality rates.^33^ These facts still do not constitute evidence to support recommending exercise to patients with small aneurysms at surveillance, since few patients have been evaluated.

A recent review included five studies and conducted a descriptive analysis.^44^ We decided that two of those studies could not be appropriately included in the systematic review without increasing clinical heterogeneity. One study had no control group,^27^ and the other evaluated respiratory physiotherapy, not exercise.^31^ Another systematic review has problems related to selection since it included the same study three times in the meta-analysis and described surrogate outcomes.^45^

The overall quality of the evidence of this review was graded “very low” because of the use of a rigorous methodology to reduce the risk of bias for clinical trials. A comprehensive and sensitive literature search was carried out, and at least two authors collected, extracted, and assessed the quality of data from studies. Additionally, a validated study was used to determine the risk of bias of the studies included.^46^ Finally, the GRADE approach was used to grade the final quality of the body of the evidence.^24^

There was heterogeneity in the amount, duration, and type of exercise among the studies included, possibly leading to different fitness levels. This heterogeneity could also lead to variation in individuals’ physiologic responses. Furthermore, the rate of loss to follow-up was high during the interventions in the clinical trials; however, this was sometimes impossible to avoid (e.g., some patients were withdrawn due to acute myocardial infarction and respiratory failure). A per-protocol analysis was thus chosen for analytic purposes. These concerns led us to conclude that the optimal duration and intensity of exercise remain undetermined. Indeed, aortic aneurysms larger than 70 mm have a lower prevalence but the worst prognosis.^47^ Thus, the evidence is not valid for this subgroup of patients.

One limitation of this review is that most of the studies included were performed in a well-controlled environment, which does not represent everyday life. Indeed, one patient in the intervention group in the study by Kothmann et al.^29^ had ventricular fibrillation and was successfully resuscitated. Aneurysms are more prevalent in men, and no study included a sufficient number of women to arrive at any conclusions for this subgroup, despite the fact that it has worse prognosis.^48^

Whether the effect of intervention is limited to the duration of exercise or can be extended even when a patient becomes sedentary later in life remains unknown. In two clinical trials, no patients were referred for surgery during surveillance, because of the short follow-up period.^18^^,^^29^ Additionally, the causes of aneurysm growth are unclear,^49^ and some rapidly expanding aneurysms reach the threshold for surgery before the expected time.^50^ This may indicate the presence of a subgroup of patients with increased exercise-related risks. Thus, to ensure safety, it is essential to set intervals for conducting ultrasound surveillance during exercise periods for patients with both small and large aneurysms.

Aneurysm diameter was imbalanced between intervention and control groups. Because aneurysm growth rate is directly dependent on original aneurysm diameter,^11^ related outcomes (e.g., aneurysm growth rate and rupture) could also be influenced.

Two-thirds of patients in the study by Myers et al.^11^ were not able to achieve the amount and intensity of exercise required for inclusion. Other types, durations, and intensities of exercise might be of value for these patients. Additionally, all studies only evaluated patients with abdominal aortic aneurysms.

There is a glaring need to perform more pragmatic clinical trials with longer follow-ups to achieve a sufficient number of patients to reduce uncertainty. Prospective studies with women are also necessary.

## CONCLUSION

The results of this systematic review and meta-analysis showed that there is very low quality evidence that exercise was effective and safe for patients with asymptomatic aortic aneurysms. Exercise did not impact aneurysm expansion rates. Six weeks of preoperative exercise decreased renal and cardiovascular surgical complications and reduced intensive care unit stays. Preoperative and postoperative forward walking reduced hospital stays. These outcomes need more studies to confirm the potential use of exercise for aortic aneurysm patients, since the quality of the evidence was judged as very low quality for all the outcomes studied.

## PERSPECTIVE

Patients with aortic aneurysms are faced with a dilemma: although exercising could increase the risk of aneurysm rupture, a sedentary lifestyle increases the risk of death, mainly due to coronary artery disease. The prevalence of aortic aneurysms is high in older patients,^3^ but most patients have small abdominal aortic aneurysms with higher mortality rates compared with patients of the same age, depending on the clinical condition.^12^ Therefore, this issue is relevant for patients, exercise professionals, and stakeholders involved in creation of new treatment interventions. To our knowledge, no other systematic review has addressed this issue with the same level of quality. This review revealed no deaths or aneurysm ruptures related to exercise. Additionally, although the clinical trials showed no reduction in aneurysm growth rates, a retrospective cohort with longer follow-up showed reductions in aneurysm growth rate and in the number of patients referred for surgery. This evidence demonstrates reductions in cardiac and renal complication rates, hospital stays, and intensive care unit stays. The present review identifies a new patient population in whom the benefits of exercise should be studied. While the general population experiences increased quality and quantity of life from exercising, patients with aneurysms might benefit from exercise as a treatment option.
